# Breast cancer in reproductive-age women in Croatia: trends, demographic shifts, and correlation with human development index

**DOI:** 10.3389/fpubh.2026.1786135

**Published:** 2026-03-05

**Authors:** Ljilja Balorda, Nataša Lisica-Šikić, Ana Balorda, Vanja Tešić

**Affiliations:** 1 Department of Social Medicine and Public Health, Institute of Public Health Zadar, Zadar, Croatia; 2 Department of Health Studies, University of Zadar, Zadar, Croatia; 3 Occupational and Sports Medicine, Disability Prevention and Psychosocial Risks in the Workplace Division, Croatian Institute of Public Health, Zagreb, Croatia; 4 Department of Epidemiology, Teaching Institute of Public Health “Dr. Andrija Štampar”, Zagreb, Croatia; 5 School of Medicine, University of Rijeka, Rijeka, Croatia

**Keywords:** breast cancer, human development index, incidence, mortality, reproductive age, trends

## Abstract

**Introduction:**

Croatia has a moderate breast cancer (BC) incidence rate and lower survival rates compared to other countries. This study examined trends in BC among women of reproductive age (15–49) in the Republic of Croatia from 2001 to 2022, with particular attention to socio-economic and demographic transition.

**Materials and methods:**

Incidence and mortality data for BC were obtained from the Croatian National Cancer Registry and the Croatian Bureau of Statistics. We used the subnational and gender-specific Human Development Index to assess regional differences and their correlation with BC. The age-standardized rate was calculated per 100.000 women aged 15 to 49 years, using direct standardization to the European Standard Population 2013, and the average annual percent change (AAPC) was determined. The mortality-to-incidence ratio (MIR) was used to monitor long-term cancer control.

**Results:**

Over 22 years in Croatia, the BC incidence rate increased, with an average of 52.1 (SD = 8.8). The rate was significantly higher in Adriatic Croatia and the City of Zagreb (*F* = 14.9, *p* < 0.001). The AAPC showed a significant upward trend in all regions. The BC mortality rate showed a decreasing trend across regions, except in the City of Zagreb, but this trend was not statistically significant. At the national level, the average mortality rate was 7.8 (SD = 1.2). There is no significant difference in mortality rates between regions (*F* = 1.936, *p* < 0.130). A significant positive correlation was found between HDI and BC incidence, and a negative correlation with MIR across all regions. The correlation with mortality was mainly negative but not significant.

**Conclusion:**

Breast cancer in this group of women is still increasing, and therefore, it is essential to implement public health policies that will improve prevention, early detection, and personalized treatment.

## 1 Introduction

Breast cancer (BC) is one of the leading causes of death among women in most countries, and incidence rates are expected to keep increasing (1, 2).

Breast cancer in women of reproductive age, although less common than in older women, demonstrates several distinct clinical and biological characteristics. In this population, tumors are more frequently associated with aggressive biological behavior, higher histological grade, and faster progression compared with postmenopausal patients (3, 4). While hormone receptor–positive tumors remain common, younger women show a relatively higher frequency of hormone receptor–negative and triple-negative subtypes, which have important therapeutic and prognostic implications (3, 4). These tumors are often genetically predisposed, and they have a higher frequency of mutations in BRCA1 and BRCA2. Due to the dense breast tissue, mammography is challenging to read, so ultrasound and breast MRI are more often used. An additional challenge is breast cancer occurring during pregnancy or lactation, when physiological changes in breast tissue may delay diagnosis and complicate clinical evaluation (5). Advances in imaging, earlier detection strategies, and treatment approaches have contributed to an improvement in survival in younger women (3, 5).

In a study about cancers in adolescents and young adults (15–39 years), BC is the most common in both incidence and mortality. Countries with a higher Human Development Index (HDI) tend to have a higher incidence, while countries with a low HDI experience higher mortality rates (6, 7). Among women of reproductive age from 1992 to 2021, BC showed a globally increasing trend of 0.47%, ranging from −0.43% in high sociodemographic index regions to 2.03% in low-middle sociodemographic index regions (8).

The Republic of Croatia is classified among countries with a moderate incidence of breast cancer, but it shows relatively high mortality rates. Although survival rates are improving, they remain among the lowest in the Southern European region (9). In 2016, Croatia had the highest BC mortality rate, 19.3 for all ages, among EU countries, and it is one of the five EU countries that did not experience a constant decline in EAPC (Estimated Annual Percent Change) mortality from 2001 to 2016 (10). According to recent studies on breast cancer in young women, Croatia ranks fifth worldwide in terms of the average annual percent change (AAPC) in incidence rates (1). Several authors recommend subnational analyses to identify regional disparities (10–14).

Croatia is also experiencing significant demographic shifts, characterized by an ageing population and a decline in population size, particularly among younger age groups (15, 16). Croatia records marked regional socioeconomic disparities, with some regions experiencing higher levels of development and faster progress than others.

Numerous studies show a correlation between breast cancer indicators and the HDI. The HDI is calculated from average years of schooling, gross national income per capita, and life expectancy (17). Countries with higher HDI scores have a higher incidence rate but a lower mortality rate (11, 14, 18–23).

An increase in the number of breast cancer cases is expected across all HDI groups; however, in countries with high and very high HDI, this increase is not anticipated among younger women (8). As Croatia belongs to the very high HDI group, a decline in incidence and mortality would be expected; however, this trend in the premenopausal women’s incidence rate has not occurred. The reasons for this discrepancy need further investigation.

BC in women of reproductive age has a broad impact, affecting not only the patients but also their children, society, employment, fertility, and the healthcare system (5, 24, 25).

It is estimated that in 2020, 1 million children worldwide were orphaned by their mother’s death due to cancer, including children under the age of 18. In 25% of cases, BC was the cause of death. This situation has a significant impact on children’s psychological and economic well-being, and in the long term, it impacts their health and educational outcomes (26).

In settings with moderate incidence, high mortality, and low survival rates, detailed analyses are required. Our research aims to analyze BC trends in reproductive-age women over 22 years of age in the Republic of Croatia to establish regional disparities and examine the correlation with HDI. The National Program for Early Detection of Breast Cancer was introduced in the Republic of Croatia in 2006, and it targets women aged 50 to 69 years (27).

According to our knowledge, this study is the first to use both subnational and gender-specific HDI in the analysis of breast cancer among women of reproductive age.

## 2 Materials and methods

In this study, we analyzed breast cancer (BC) trends among women of reproductive age, defined by the World Health Organization as those aged 15 to 49 years (28). Data on incidence and mortality were obtained from the Croatian Cancer Registry of the Croatian Institute of Public Health and the Croatian Bureau of Statistics, with appropriate permissions for their use. Mortality data for malignant neoplasm of the breast, International Classification of Diseases 10th revision (ICD-10) code C50, were obtained for this population group from the Croatian Bureau of Statistics, along with mid-year population estimates for women (29). Incidence data for C50 were collected from the Croatian Cancer Registry. All data were provided for each year from 2001 to 2022 and were grouped into 5-year intervals. All data were aggregated and contained no personal identifiers; therefore, ethical approval was not required.

We calculated the mortality-to-incidence ratio (MIR) from the data obtained, which shows cancer trends and the effectiveness of cancer control (30, 31). Some studies demonstrated that it correlates well with five-year survival in women with breast cancer and can be a helpful measure for monitoring and analyzing geographic and racial differences in BC patients (30).

We used the Subnational Human Development Index (SHDI) and the gender-specific HDI for women, as subnational and gender differences existed in income, education, and life expectancy (32, 33). Data were provided for each year from 2001 to 2022. This data is available on Global Data Lab (Global Data Lab, 2025) (34).

The Human Development Index (HDI) is a composite measure in three dimensions of human development: health (life expectancy at birth), education (mean and expected years of schooling), and gross national income per capita. HDI values range from 0 to 1, and they are commonly categorized into four levels: low (<0.550), medium (0.550–0.699), high (0.700–0.799), and very high (≥0.800) (35). HDI has been shown to correlate with BC incidence and mortality, with countries of higher HDI having higher incidence but lower mortality rates (11, 21, 22, 36). Examining the HDI at the subnational and gender-specific levels can clarify regional inequities in disease burden in BC.

Croatia is divided into four regions according to the “Statistical classification of spatial units of the Republic of Croatia, NUTS 2 classification”: Adriatic, North, Pannonian, and the City of Zagreb as a separate region (37). All data will be presented at both the national and NUTS 2 regional levels.

### 2.1 Statistical analyses

In our research, we used descriptive statistics, and data were presented as absolute numbers and as truncated age-standardized incidence rates (ASIR) and age-standardized mortality rates (ASMR) per 100,000 women, using direct standardization to the European Standard Population 2013. The mortality-to-incidence ratio (MIR) was used to monitor the long-term control of BC. To test subnational differences, we used the analysis of variance (ANOVA). The Kolmogorov–Smirnov test was used to assess the distribution of the data, and the Levene test was used to determine homogeneity of variance. When data did not meet this criterion, we used the Kruskal-Wallis ANOVA test to establish subnational differences. To examine trends in age-standardized BC incidence and mortality rates, we used Joinpoint regression analysis (Joinpoint Regression Program, version 5.3.0, National Cancer Institute, Bethesda). The average annual percent change (AAPC) with a 95% confidence interval (CI) was calculated. The AAPC provides a better description of trends when data are non-linear and can be used to characterize shorter segments or when data are sparse or derived from small geographic areas (38). The correlation coefficients (r) between the independent variable, the HDI, and the dependent variables ASIR, ASMR, and MIR were calculated. We use linear regression analysis to assess the strength of the correlation and report the regression coefficient (b) and the correlation coefficient (R). The proportion of variance explained by HDI was assessed using the coefficient of determination (R^2^). The linearity and homoscedasticity of the data were evaluated by residual analysis. The Durbin-Watson test was used to determine the presence of autocorrelation in the time-series residuals. When the Durbin–Watson statistic was below 1.5 or above 2.5, a lag-1 transformation was used to remove first-order autocorrelation. The data were transformed by applying a lag-t-1 transformation to the dependent variables (ASIR, ASMR, MIR), creating an additional variable called lag for each year t, that represents the values from the previous year (Y_{t-1}). A *p*-value of <0.05 was considered statistically significant. Statistical analyses were performed using Statistica software (version 14.0.1; StatSoft Europe) (39). Tables and graphs were created with Microsoft Excel (Microsoft Corporation, Redmond, WA, USA).

The number of statistical tests performed increases the risk of a Type I error, and no correction for multiple comparisons was applied. Therefore, our results are interpreted with caution, emphasizing effect sizes, confidence intervals, and consistency across regions and outcomes.

## 3 Results

### 3.1 Population

According to data from the Croatian Bureau of Statistics, Croatia is facing negative demographic trends. The female population declined by 10.7%, from 2,233,432 in 2001 to 1,995,130 in 2022 (19).

Within the reproductive-age group (15–49 years), the decline was more pronounced, with a 23% reduction (from 1,035,748 in 2001 to 794,595 in 2022). Regionally, the most significant decrease was observed in Pannonian Croatia (−36%, −109,602), followed by Northern Croatia (−21%, −43,165), Adriatic Croatia (−20%, −65,104), and the City of Zagreb (−12%, −23,282) (19).

### 3.2 Human development index

**Figure 1:**
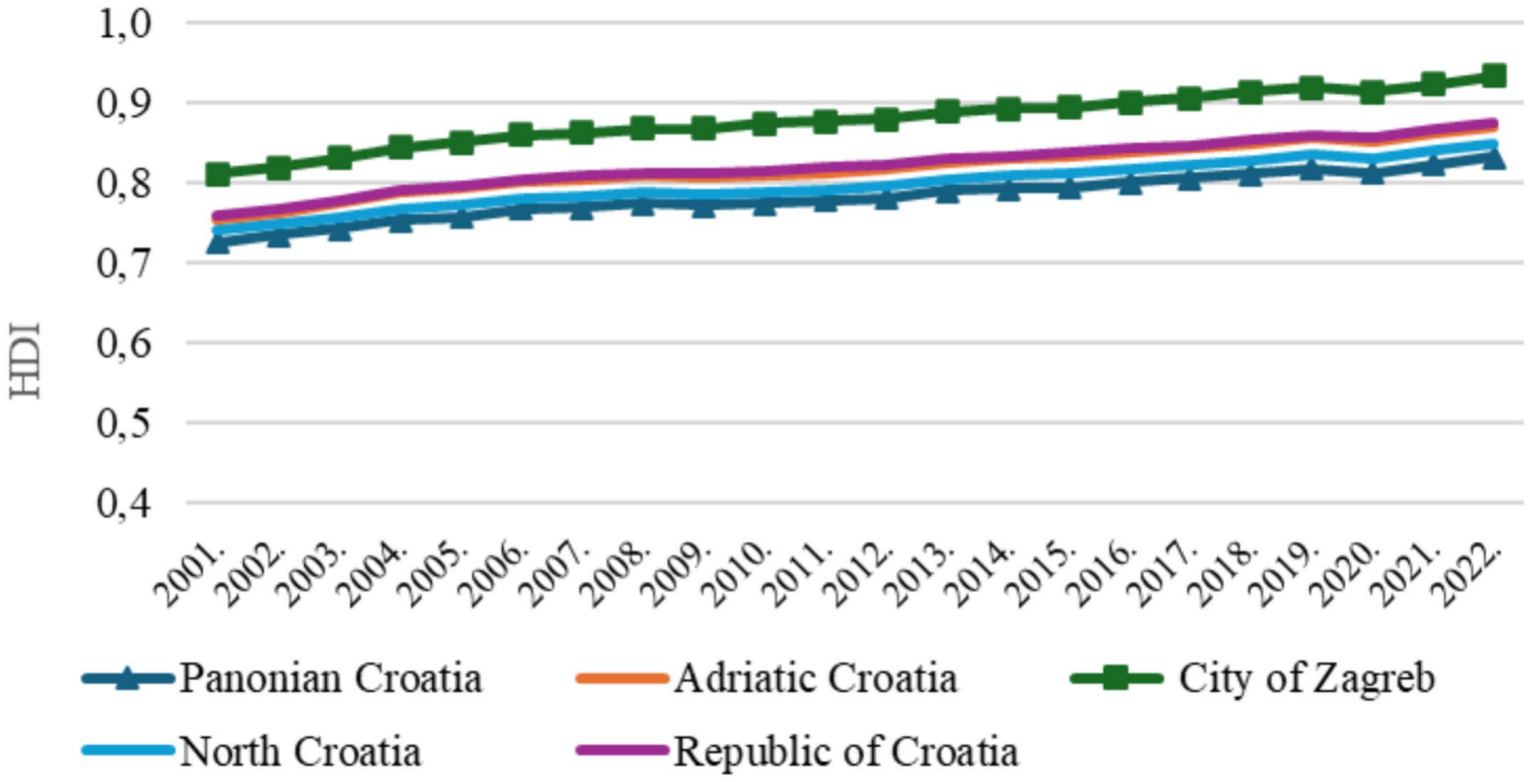
Human development index in Croatia.

Croatia and its four subnational regions all showed an upward trend in the Human Development Index (HDI) from 2001 to 2022. Among them, only the City of Zagreb maintained a very high HDI level throughout the entire period. At the national level, Croatia shifted from a high to a very high HDI category in 2006. The same change occurred in Adriatic Croatia in 2006, Northern Croatia in 2013, and Pannonian Croatia in 2016 (Figure 1).

The results revealed a significant difference in HDI across subnational regions, *F* (3, 84) = 40.91, *p* < 0.001. *Post hoc* tests showed that the City of Zagreb was significantly different from all the other regions (p < 0.001). A significant difference was also found between Pannonian and Adriatic Croatia (*p* = 0.002). No statistically significant differences were observed between Pannonian and Northern Croatia (*p* = 0.388), nor between Adriatic and Northern Croatia (*p* = 0.142).

### 3.3 Breast cancer in reproductive-age women

A total of 10,695 new cases of breast cancer (BC) were diagnosed among women of reproductive age in Croatia between 2001 and 2022. Of these, 37% (3,936) occurred in Adriatic Croatia, 24% (2,564) in Pannonian Croatia, 23% (2,429) in the City of Zagreb, and 16% (1,766) in North Croatia. During the same period, 1,683 women of reproductive age died from BC. The regional distribution of deaths was as follows: 33% (544) in Adriatic Croatia, 29% (474) in Pannonian Croatia, 21% (335) in the City of Zagreb, and 17% (285) in North Croatia. Throughout the entire period, women of reproductive age made up 18.6% of all BC cases and 8.5% of BC-related deaths.

**Figure 2:**
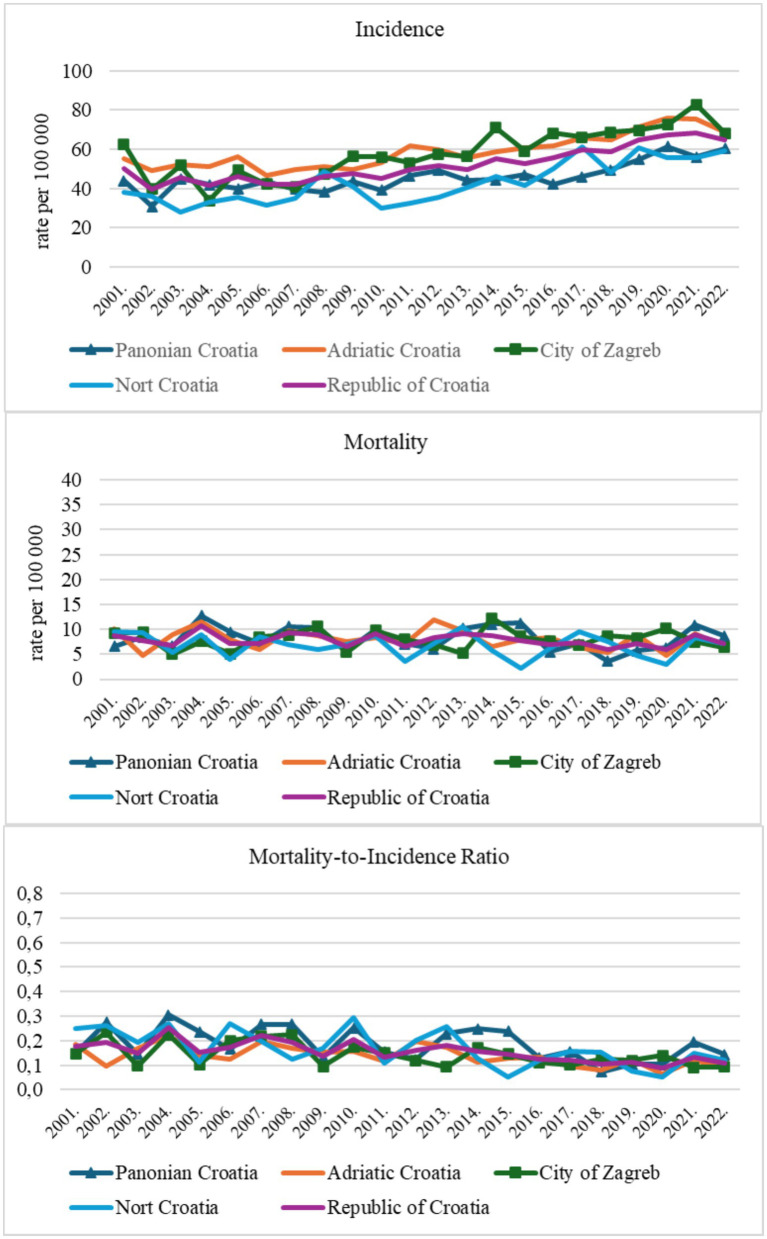
Breast cancer in women of reproductive age in Croatia **(a)** incidence, **(b)** mortality, and **(c)** mortality-to-incidence ratio.

Croatia had an increasing trend in age-standardized incidence rate (ASIR) (M = 52.1, SD = 8.8; average annual percent change (AAPC) = 1.9*, 95% CI = 1.4–2.6). The difference across subnational regions in ASIR was significant, H (3, N = 88) = 30.95, *p* < 0.001. *Post hoc* analysis of mean ranks showed that Adriatic Croatia (61.32) and the City of Zagreb (57.68) had higher incidence rates than Panonian Croatia (31.91) and North Croatia (27.09; all *p* < 0.01). There were no differences between the City of Zagreb and the Adriatic area, nor between North and Panonian Croatia (*p* = 1.000). The AAPC indicated a statistically significant upward trend in all regions (Tables 1, 2; Figure 2a).

**Table 1:** Breast cancer among reproductive-age women in Croatia (2001–2022).

Incidence				
Region	Mean	SD	AAPC	95% CI
Republic of Croatia	52.1	8.8	1.9*	1.4 to 2.6
Pannonian Croatia	45.9	7.2	2.1*	1.1 to 3.0
Adriatic Croatia	59.0	8.6	1.6*	1.1 to 2.4
City of Zagreb	58.0	12.5	1.6*	0.6 to 3.3
North Croatia	43.0	10.6	3.1*	1.9 to 4.4
Mortality				
Republic of Croatia	7.8	1.2	−0.8	−2.6 to 0.9
Pannonian Croatia	8.2	2.4	−0.6	−3.6 to 2.1
Adriatic Croatia	8.0	2.0	−1.1	−3.1 to 0.8
City of Zagreb	8.0	1.9	0.1	−1.9 to 2.1
North Croatia	6.8	2.3	−1	−3.9 to 1.8
Mortality-to-incidence ratio				
Republic of Croatia	0.16	0.04	−3.0*	−4.7 to −1.5
Pannonian Croatia	0.19	0.07	−2.5	−6.2 to 0.9
Adriatic Croatia	0.14	0.04	−2.8*	−5.0 to −0.6
City of Zagreb	0.14	0.05	−2.8*	−5.3 to −0.3
North Croatia	0.17	0.07	−3.6*	−6.6 to −0.7

*statistically significant; SD, standard deviation; AAPC, average annual percent change; CI, confidence interval.

**Table 2:** Trends in breast cancer incidence among women of reproductive age in Croatia (2001–2022).

	Trend 1			Trend 2		
Region	Period	APC1	95% CI	Period	APC2	95% CI
Republic of Croatia	2001–2006	−1.8	−9.6 to 1.6	2006–2022	3,1*	2.5 to 4.7
Pannonian Croatia	2001–2016	0.9	−5.2 to 3.9	2016–2022	5,2*	1.6 to 17.2
Adriatic Croatia	2001–2007	−1.1	−8.6 to 1.8	2007–2022	2,7*	2.0 to 6.7
City of Zagreb	2001–2004	−9.7	−23 to 2.6	2004–2022	3,6*	2.3 to 9.7
North Croatia	/			/		

*statistically significant; APC, annual percent change; CI, confidence interval.

Croatia showed a decreasing trend in age-standardized mortality rate (ASMR) (M = 7.8, SD = 1.2; AAPC = −0.8, 95% CI = −2.6 to 0.9). All subnational regions showed a decreasing trend in BC mortality over the study period, except for the City of Zagreb; however, the AAPC was not significant in any subnational region. The BC ASMR was highest in Pannonian Croatia, with an average age-standardized rate of 8.2 (SD = 2.4), and lowest in Northern Croatia, with a mean rate of 6.8 (SD = 2.3) (Table 1; Figure 2b). The differences in ASMR across subnational regions were not statistically significant, *F* (3, 84) = 1.94, *p* < 0.130.

The Mortality-to-Incidence Ratio (MIR) on the national level showed a decreasing trend (M = 0.16, SD = 0.04; AAPC = −3.0, 95% CI = −4.7 to −1.5). All four subnational regions demonstrated a negative downward trend. Significant decreases were observed in three regions: the City of Zagreb, North Croatia, and the Adriatic region, while the trend in the Pannonian region was not statistically significant. MIR score distributions were similar across groups (Table 1; Figure 2c). The differences in MIR across subnational regions were not statistically significant, H (3, N = 88) = 7.52, *p* = 0.057. Average ranks suggested that Pannonian Croatia (54.9) and North Croatia (48.3) tended to have higher MIR values than the Adriatic region (37.4) and the City of Zagreb (37.4). *Post hoc* tests found no statistically significant differences between any pair of regions (all *p* > 0.05).

Joinpoint analysis showed distinct temporal trends: at the national level, after a decline from 2001 to 2006, a significant upward trend was observed in subsequent years. Similar patterns appeared in Pannonian Croatia after 2016, in Adriatic Croatia after 2006, and in the City of Zagreb after 2004; no temporal trends were seen in Northern Croatia (Table 2).

Statistically significant joinpoints were found only for incidence; therefore, Table 2 presents two separate trends (Trend 1 and Trend 2) with their respective APC1 and APC2 values. No statistically significant joinpoints were detected for ASMR or MIR, indicating a consistent trend over the entire study period.

### 3.4 Correlation with the human development index

A strong positive correlation was observed between HDI and ASIR at the national level (r_s_ = 0.88, *p* < 0.001) and across all subnational regions. Regression coefficients are positive at the national level (*p* = 0.001), explaining 89% of the variance (R^2^ = 0.89). At the subnational level, the relationship remains strong. These findings were also supported by Kendall’s tau coefficients (p < 0.001). A weak negative correlation between HDI and ASMR was observed; however, it was not statistically significant at either the national or subnational levels (all *p* > 0.05). The regression coefficients were mostly small and negative, except for the City of Zagreb. A significant negative correlation was found between HDI and MIR at both the national level and across all subnational regions. Linear regression indicated that HDI explained 45% of the variance (R^2^ = 0.45) at the national level. All four regions showed significant, moderate-to-strong negative correlations at the subnational level (all *p* < 0.05) (Tables 3, 4).

**Table 3:** Breast cancer in reproductive-age women, HDI correlation (2001–2022).

HDI correlation						
Incidence						
	r_s_	t(N-2)	*p*	*τ*	*Z*	*P*
Republic of Croatia	0,88	8,30	0,000	0,75	4,88	0,000
Adriatic Croatia	0,86	7,50	0,000	0,68	4,43	0,000
Pannonian Croatia	0,75	5,01	0,000	0,56	3,64	0,000
City of Zagreb	0,81	6,18	0,000	0,65	4,20	0,000
North Croatia	0,78	5,51	0,000	0,59	3,86	0,000
Mortality						
Republic of Croatia	−0,25	−1,13	0,270	−0,21	−1,36	0,175
Adriatic Croatia	−0,25	−1,17	0,257	−0,20	−1,33	0,185
Pannonian Croatia	−0,15	−0,66	0,519	−0,08	−0,54	0,592
City of Zagreb	−0,02	−0,07	0,942	−0,01	−0,08	0,933
North Croatia	−0,20	−0,92	0,370	−0,16	−1,02	0,309
Mortality-to-incidence ratio						
Republic of Croatia	−0,74	−4,92	0,000	−0,56	−3,64	0,000
Adriatic Croatia	−0,59	−3,28	0,004	−0,43	−2,79	0,005
Pannonian Croatia	−0,49	−2,49	0,022	−0,35	−2,28	0,022
City of Zagreb	−0,50	−2,57	0,018	−0,39	−2,57	0,010
North Croatia	−0,58	−3,15	0,005	−0,39	−2,51	0,012

Spearman correlations, r_s_; Kendall tau correlations, τ.

**Table 4:** Breast cancer in reproductive-age women, HDI linear regression (2001–2022).

HDI linear regression						
Incidence						
	*b*	*SE*	*t*	*p*	*R*	*R* ^2^
Republic of Croatia	195,9	46,9	4,2	0,001	0,94	0,89
Adriatic Croatia	143,7	55,6	2,6	0,019	0,90	0,81
Pannonian Croatia	189,7	36,9	5,1	0,000	0,75	0,57
City of Zagreb	270,9	56,2	4,8	0,000	0,73	0,54
North Croatia	268,7	73,6	3,7	0,002	0,86	0,74
Mortality						
Republic of Croatia	−13,9	9,7	−1,4	0,169	0,38	0,15
Adriatic Croatia	−15,3	13,6	−1,1	0,276	0,24	0,06
Pannonian Croatia	−10,4	18,5	−0,6	0,578	0,13	0,02
City of Zagreb	1,6	12,6	0,1	0,900	0,03	0,00
North Croatia	−18,0	17,0	−1,1	0,303	0,23	0,05
Mortality-to-incidence ratio						
Republic of Croatia	−0,9	0,2	−4,0	0,001	0,67	0,45
Adriatic Croatia	−0,7	0,2	−2,9	0,010	0,54	0,29
Pannonian Croatia	−1,0	0,5	−2,1	0,048	0,43	0,18
City of Zagreb	−1,2	0,3	−3,5	0,003	0,64	0,41
North Croatia	−1,9	0,6	−3,1	0,006	0,61	0,37

b, regression coefficient; SE, standard error; R, correlation coefficient; R^2^, coefficient of determination.

## 4 Discussion

Breast cancer (BC) is one of the leading causes of death among women in most countries, and incidence rates are expected to keep increasing (1, 2). In women of reproductive age, it is less common than in older women, and demonstrates several distinct clinical and biological characteristics: aggressive biological behavior, higher histological grade, and faster progression, compared with postmenopausal patients (3, 4). While hormone receptor–positive tumors remain common, younger women show a relatively higher frequency of hormone receptor–negative and triple-negative subtypes, which have important therapeutic and prognostic implications (3, 4). These tumors are often genetically predisposed, and they have a higher frequency of mutations in BRCA1 and BRCA2. Due to dense breast tissue, physiological changes may delay diagnosis and complicate clinical evaluation (3, 5).

Young women are often underrepresented in research. They tend to have a more aggressive BC phenotype, with a higher proportion of grade 3 tumors, triple-negative status, and human epidermal growth factor receptor 2 overexpression. They also exhibit lympho vascular invasion, lymphocytic infiltration, and poorer outcomes at any stage at diagnosis (5, 40).

Croatia has a moderate BC incidence rate and lower survival rates than those in other countries. In this context, we analyzed trends in BC among women of reproductive age (15–49) in the Republic of Croatia from 2001 to 2022, with attention to socioeconomic and demographic transitions and regional differences.

The results showed that ASIR increased continuously and varied significantly across regions. Adriatic Croatia and the City of Zagreb had higher incidence rates than Pannonia and Northern Croatia. Additionally, Adriatic Croatia and the City of Zagreb exhibited higher HDI values. We found a strong positive correlation between ASIR and HDI. ASMR decreased; rates did not differ significantly across subnational regions, and the correlation with the HDI was mainly negative and weak. The MIR showed a decreasing trend and also did not differ significantly between regions. The MIR had a significant negative correlation with HDI. The correlation was from moderate to strong. Due to increasing HDI and improvements in healthcare, clinical protocols, and early diagnosis, survival has improved across all subnational regions.

In countries with low HDI, 49% of women with BC are in premenopausal age, and in very high-HDI countries, 21% BC are in premenopausal age (41). In very high-income countries, incidence has a decreasing trend (8), but some high-income countries, such as France, Italy, New Zealand, and Norway, report an increasing incidence of premenopausal BC, and it is probably due to demographic changes (5). In Croatia, the data show an even lower percentage in 2022 (17%), but for the period from 2001 to 2022, it was 18.6%. This data suggests that demographic changes may be responsible for a lower proportion of women of reproductive age among all women with BC. During the observed period, our data revealed a strong demographic and socioeconomic transition in Croatia. There was a 23% decrease in the population of reproductive-age women at the national level. The changes are more pronounced in Pannonian Croatia (−36%) and North Croatia (−21%). These two regions had a lower HDI compared with the Adriatic region and the City of Zagreb during the observed period. The City of Zagreb had a significantly higher HDI than the other three regions, with a rising ASIR and mortality remaining positive, with an AAPC of 0.1; however, the 95% CI was −1.9 to 2.1. This is important for further research.

Due to the migration of younger populations, less developed regions with lower HDI might experience slower future improvements in HDI. This can lead to decreased investment in medical equipment and health worker education, resulting in poorer health coverage and making these areas less attractive to younger health workers, which further reduces the quality of care. We did not include data on regional healthcare workforce distribution or diagnostic intervals because data were unavailable for the entire study period in these regions. Future research should include such data to better assess their potential impact on regional disparities in cancer outcomes and healthcare access for women.

At the national level, we observed that, following the HDI’s shift from high to very high in 2006, ASIR among women of reproductive age increased notably thereafter. From 2001 to 2006, the annual percent change (APC) in BC among reproductive-age women was −1.8; from 2006 to 2022, the APC increased to 3.1, which was statistically significant. However, AAPC for the entire period was 1.9, which was also statistically significant. Similar patterns were observed across subnational regions. Once the HDI reached very high levels, the incidence rate of BC subsequently increased.

A statistically significant higher incidence rate was observed in the Adriatic and in the City of Zagreb (ASIRs of 59 and 58 per 100,000 women, respectively). Previous research has revealed a similar pattern, but some studies report that, in very high-income countries, the incidence decreased among premenopausal women. A study that analyzed BC incidence in reproductive-aged women from 1992 to 2021 globally showed an increasing trend with a 0.47% annual rise. High SDI countries had an ASIR of 47, whereas the global ASIR was 27.5. The highest rates were Monaco (109) and Panama (67.9). Turkey had the highest EAPC (8.7%) for BC incidence. In another study from 1990 to 2021, similar results were observed: high-SDI countries showed a decreasing trend. Western Europe had ASIR 51.2 in 1990, and 46.1 in 2021; EAPC was −0.3. Eastern Europe had ASIR 29.9 in 1990, and 28.9 in 2021; EAPC was −0.5 (42). Our study found that ASIR in Croatian women of reproductive age was more similar to Western Europe, but in Croatia, rates still showed an increasing trend.

Research from 1998 to 2012 showed that mortality rates in premenopausal women were globally higher in low HDI countries (8.5), and lower in very high and medium HDI countries, ranging from 3.3 to 4.5 (11). Similar results were obtained in another study (1990 to 2021) that analyzed the influence of the health system on mortality rates; only countries with advanced health systems showed a decreasing mortality trend (43). In Europe, from 2015 to 2019, the ASMR for women aged 20–49 was 6.5 (44). Eastern Europe had an ASMR of 9.6 in 1990 and 6.2 in 2021; the EAPC was −1.95 for the period 1990–2021. Meanwhile, Western Europe had an ASMR of 10.8 in 1990 and 5.2 in 2021; the EAPC was −2.4 (42). Our research revealed a higher mortality rate of 7.8, similar to that of high-developed countries since the 2000s. However, rates are decreasing at both the national and subnational levels, except in the City of Zagreb. The AAPC for the City of Zagreb was 0.1 (95% CI: −1.9 to 2.1). The lowest rate was in North Croatia, and the highest was in Pannonian Croatia.

In some studies, in addition to incidence and mortality rates, MIR was also examined. MIR is a practical measure for evaluating long-term cancer follow-up and the effectiveness of cancer control programs. All these studies showed a strong negative correlation of HDI with the MIR of BC (11, 14, 45, 46). In some studies, instead of MIR, the case fatality percentage (CF) is calculated as ASMR divided by ASIR, expressed as a percentage. MIR decreases as the HDI level increases. For premenopausal women, case fatality ranged from 10.8% in very high HDI countries to 47.0% in low HDI countries. North America, New Zealand, and the northern, southern, and western parts of Europe had less than 10% (11). In other research, MIR was used as an indicator of BC care quality, and the global BC MIR in 2020 for all age groups was 0.30. It was the highest in Africa (0.48), the lowest in North America (0.17), and 0.27 in Europe (45). Our research showed a significant decline in MIR across all regions except the Pannonian region. The average MIR at the national level for the reproductive age group was 0.16, similar to rates observed in high-developed countries.

The correlation between the HDI and BC incidence rates was statistically significant at the national level and across all four regions, whereas no significant correlation was observed with mortality rates. A Norwegian study on BC in premenopausal women showed that 5-year relative survival improved only in women with high socioeconomic status in both regional and distant disease, but not in women with low socioeconomic status, even with universal health care (47). The Republic of Croatia has universal health care, but significant demographic changes, reflected in the population decline, especially among women of reproductive age in the Adriatic and Pannonian regions, could also affect the number of highly educated health workers. In the future, this could contribute to diagnostic delays.

In our research, we did not find a statistically significant decrease in the mortality rate, and the correlation with the HDI was very weak. Due to higher risks of aggressive BC subtypes in reproductive-age women, further investigation should be conducted, and an analysis of regional differences according to risk factors for BC in reproductive-age women should be performed.

Health inequalities in cancer care are most often caused by the complex interaction of health literacy, socioeconomic status, age, geographic access to services, and lifestyle-related factors, which together influence cancer prevention, diagnostic delays, and outcomes (48).

According to the ESO-EMSO 2022 (European School of Oncology-European Society of Medical Oncology), managing lifestyle-modifiable risk factors for BC is also important, including maintaining a healthy body weight, physical activity, and limiting alcohol consumption and smoking. Some studies show that women with obesity at a premenopausal age have a lower risk for BC. Still, a higher waist-hip ratio is associated with increased risk for BC in premenopausal women, as well as high energy-rich foods and ultraprocessed foods (5, 49, 50). High mammographic density is a risk factor across all age groups, and an unhealthy diet contributes to it (49). Some studies have found that reproductive patterns also affect BC burden, including early menarche, fewer children per woman, later age at first birth, and extended use of oral contraception (49).

In all ages, but especially among young women, cancer has an impact not only on individual health and well-being, but it also has a strong influence on the quality of life and income, negatively influences a person’s work life, leading to part-time work, unemployment, and early retirement, and reduces opportunities for education and training. Cancer also affects mental health and can lead to stress, anxiety, and depression (24). Population-based education is necessary at both the national and subnational levels. Still, personalized approaches to lifestyle counseling are essential due to the transition to sedentary lifestyles, urbanization, and unhealthy dietary habits.

In countries with higher HDI, women aged 15–54 have three to four times higher risks of cancer (all sites) than in countries with lower HDI (26). A multidisciplinary approach is essential to improve survival rates among this group of women. The healthcare system needs to ensure the availability of diverse healthcare professionals, including surgeons, oncologists, gynecologists, community nurses, genetic testing specialists, and psychologists, given the effects on the quality of life and fertility (5).

The importance of this research lies in its 22-year subnational analysis, which investigates differences in incidence and mortality rates among women of reproductive age during the demographic transition and within the context of the HDI at both subnational and gender-specific levels. This age group is often underrepresented in BC studies.

A limitation of this study is that we did not analyze the BC stage and subtypes, which should be addressed in future research among women of reproductive age. Previous studies confirm that genetic influences are significant in BC among women of reproductive age; however, physical activity, alcohol consumption, and dietary habits are also important, and these risk factors are modifiable. Higher rates of premenopausal abdominal obesity, measured as a higher waist-hip ratio, and lower rates of breastfeeding are linked to a higher proportion of triple-negative BC (51). Oral contraception use also influences BC with differences in molecular subtypes in premenopausal women; the risk varies by subtype, with a significant increase in ER-negative breast cancer and a non-significant increase in triple-negative BC (52). Future studies at the subnational level should also include analyses of lifestyle risk factors and reproductive patterns, which could help reduce the burden of BC.

## 5 Conclusion

This analysis has examined regional differences in three breast cancer indicators among women of reproductive age in Croatia. A positive correlation has been observed between HDI and incidence, and a negative correlation with MIR, but no significant negative correlation with mortality. The results showed that ASIR continued to increase and varied significantly across regions. In the future, due to negative demographic trends and lifestyle changes, we might also expect an influence on ASMR and MIR.

Awareness of breast cancer burden among reproductive-age women should be raised in public health policies on national and regional levels to improve early detection and survival rates. Additionally, health literacy and education about increasing risk factors among reproductive-age women should be implemented.

## Data Availability

The raw data supporting the conclusions of this article will be made available by the authors, without undue reservation.
