# Supplementary figures and images for: Breast cancer in reproductive-age women in Croatia: trends, demographic shifts, and correlation with human development index
Source: Front Public Health. 2026 Mar 5;14:1786135. doi: 10.3389/fpubh.2026.1786135 (PMC12999559; doi:10.3389/fpubh.2026.1786135)

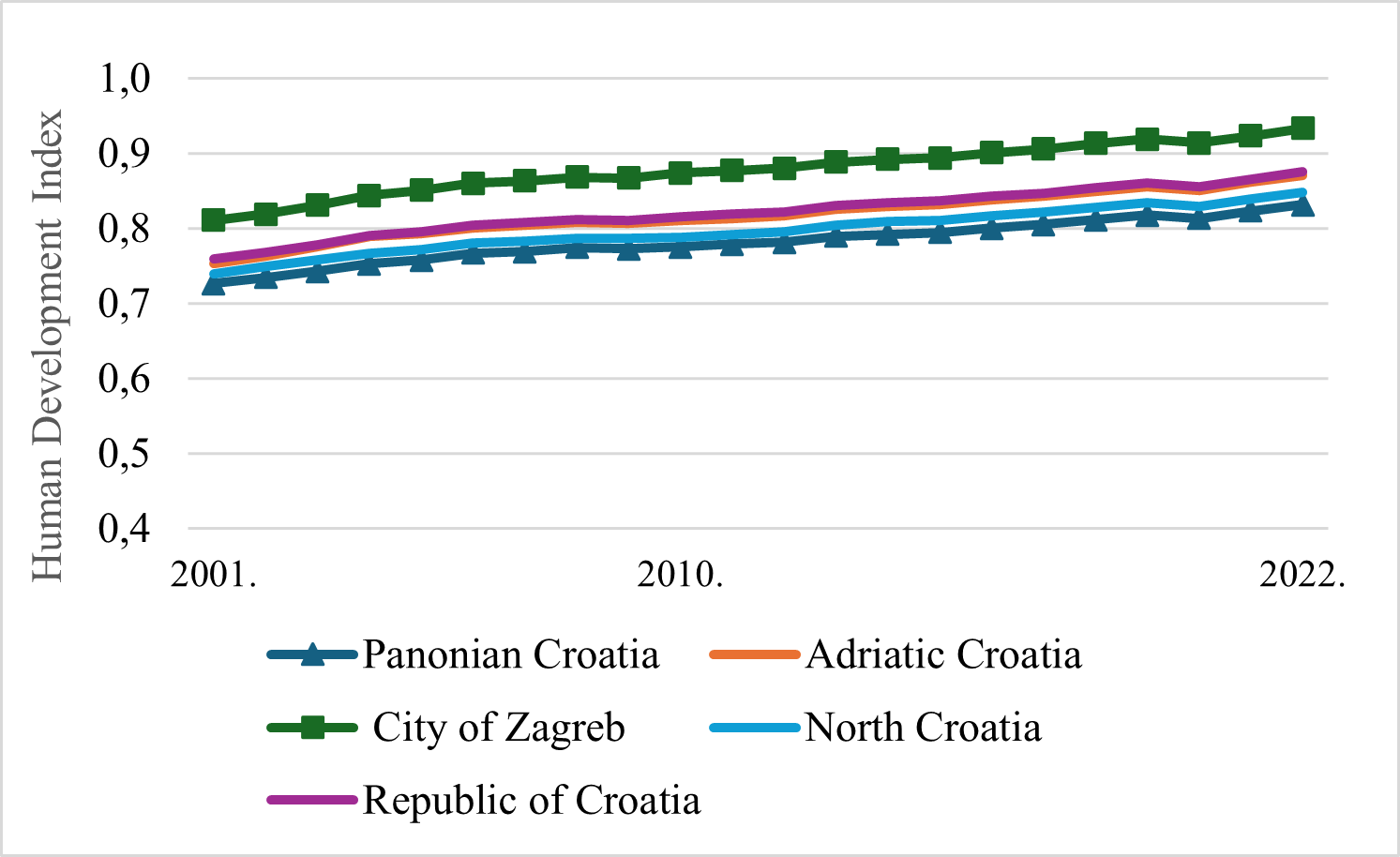

Supplement: Supplementary file 1 [file Image_1.png]

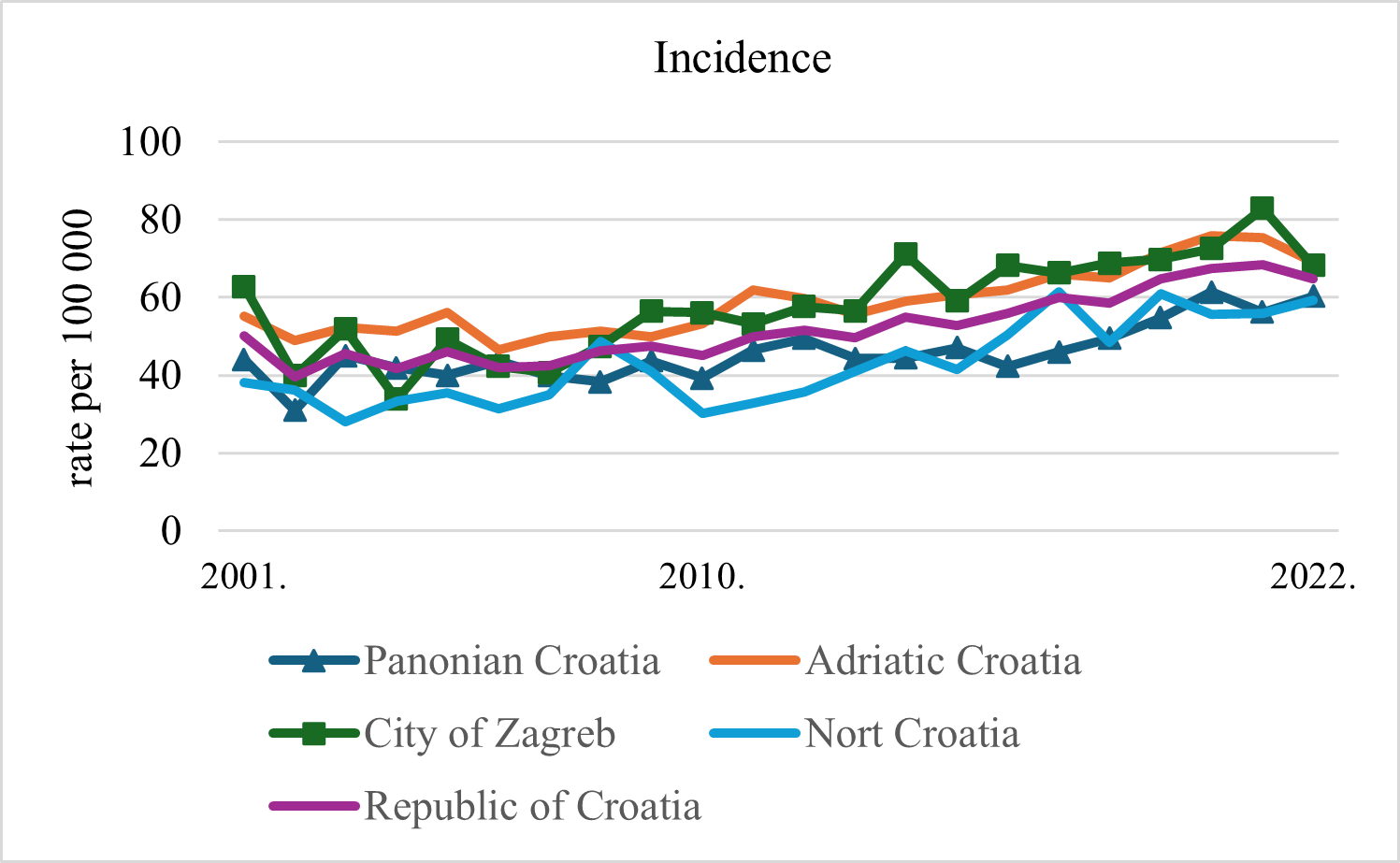

Supplement: Supplementary file 2 [file Image_2.png]

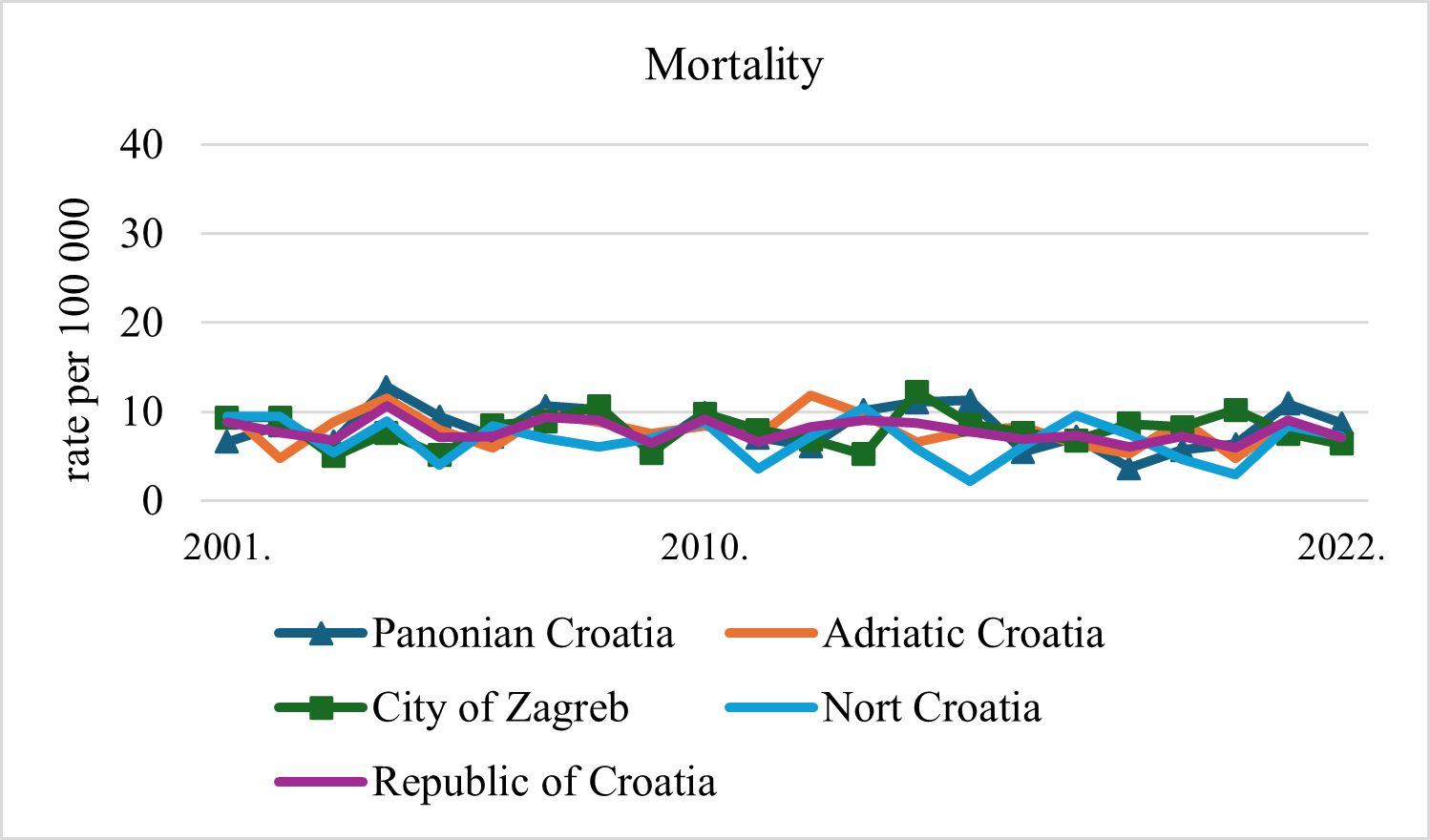

Supplement: Supplementary file 3 [file Image_3.png]

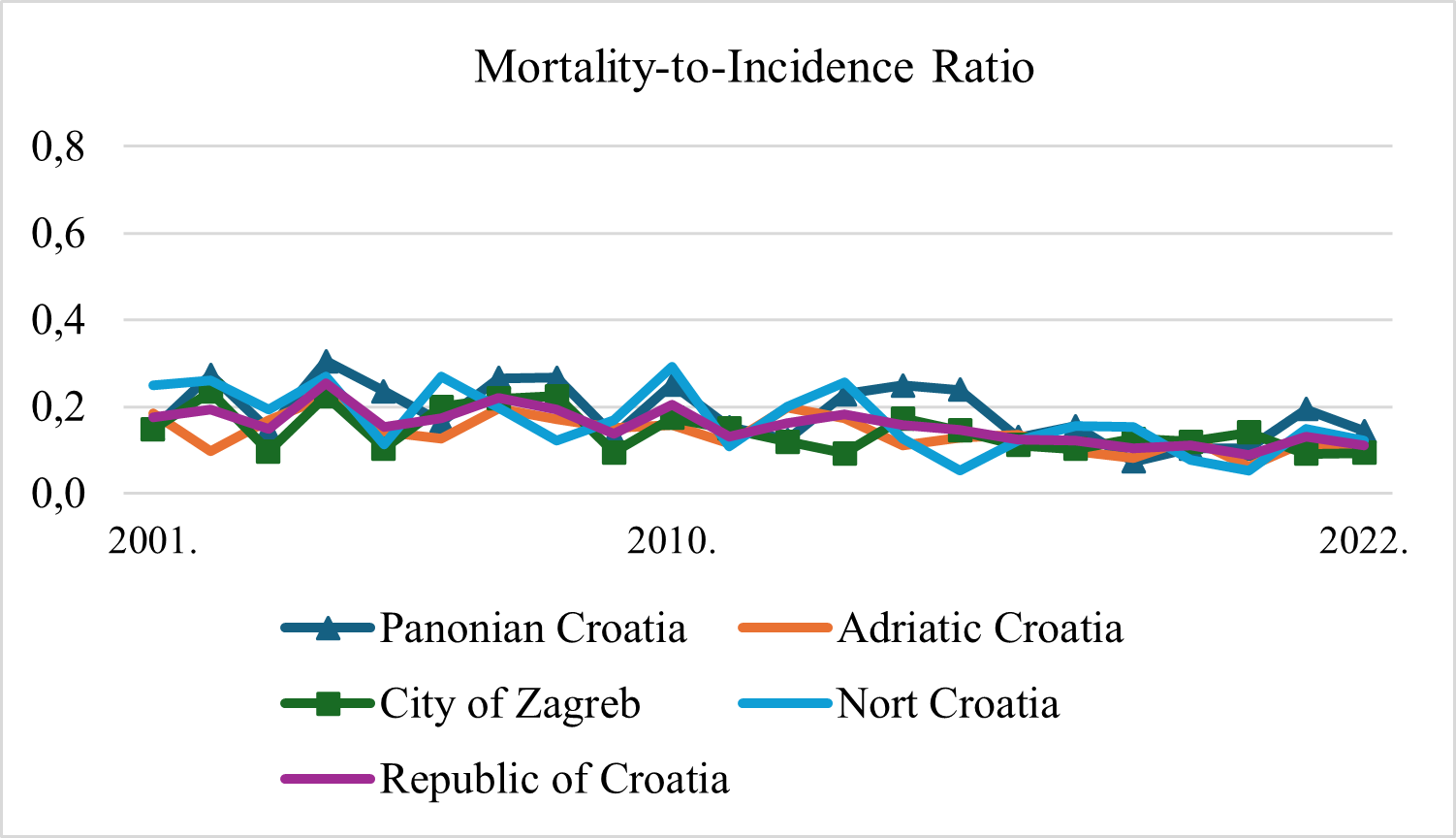

Supplement: Supplementary file 4 [file Image_4.png]
